# Active Vitamin D and Vitamin D Receptor Help Prevent High Glucose Induced Oxidative Stress of Renal Tubular Cells via AKT/UCP2 Signaling Pathway

**DOI:** 10.1155/2019/9013904

**Published:** 2019-05-28

**Authors:** XiaoJuan Zhu, ShengHua Wu, HanCheng Guo

**Affiliations:** ^1^Department of Nephrology, Zhongshan Hospital, Xiamen University, Xiamen, Fujian 361004, China; ^2^Department of Emergency, Zhongshan Hospital, Xiamen University, Xiamen, Fujian 361004, China

## Abstract

**Background:**

It has been documented that vitamin D supplementation showed an improvement of symptoms of diabetic nephropathy; however, the underlying mechanisms remain unknown. We here tested the hypothesis that active vitamin D is able to up-regulate AKT/UCP2 signaling to alleviate oxidative stress of renal tubular cell line HK2.

**Methods:**

There are eight groups in the present study: normal glucose, osmotic control (5.5 mmol/L D-glucose+24.5 mmol/L D-mannitol), NAC control (30 mmol/L D-glucose + 1.0 mmol/L N-Methylcysteine), high glucose, high glucose+VD, high glucose (HG)+VD+siVDR, HG+VD+AKT inhibitor (AI), and high glucose+VD+UCP2 inhibitor (Gelipin). Concentration of superoxide dismutase (SOD) and malondialdehyde (MDA) was analyzed by ELISA. Reactive oxygen species (ROS), mitochondrial membrane potential and apoptosis were measured by flow cytometry. JC-1 was evaluated by flow cytometry. The presence of VDR, AKT, and UCP2 in HK cells was assessed using RT-PCR and western blot analyses.

**Results:**

VD administration significantly upregulated the SOD activation and downregulated MDA levels compared to HG group. siVDR, AKT inhibitor, and UCP2 inhibitor significantly suppressed the activation of SOD and increased the expression of MDA compared to VD group. ROS generation and apoptosis of HK2 cells in HG+VD group were significantly lower than those in HG, HG+VD+siVDR, HG+VD+AI, and HG+VD+Gelipin group. ΔΨm in HG+VD group was obviously higher than those in HG, HG+VD+siVDR, HG+VD+AI, and HG+VD+Gelipin group. Decreased mRNA and protein levels of VDR, p-AKT, and UCP2 were observed in HG+VD+siVDR, HG+VD+AI, and HG+VD+Gelipin group compared to those in HG+VD group.

**Conclusions:**

siVDR, AKT inhibitor, and UCP2 inhibitor elevated the ROS and apoptosis of HK2 cells while attenuating the mitochondrial membrane potential, suggesting that vitamin D protects renal tubular cell from high glucose by AKT/UCP2 signaling pathway.

## 1. Introduction

Diabetic nephropathy (DN) is featured as the leading cause of end-stage renal disease in foreign countries and ranked as the second common cause of renal failure in China. Vitamin D deficiency is involved in a decreased insulin resistance, type 2 diabetes mellitus, and a higher risk of DN. Lower 25-hydroxyvitamin D [25(OH)D] in circulation is related to elevated renin-angiotensin system (RAS) activity and blood pressure. The use of vitamin D as supplementary in chronic kidney diseases [1] has been long reported. Factors activating the intrarenal RAS eventually lead to diabetic nephropathy, such as high glucose intake. Recent researches showed high glucose would induce apoptosis in human proximal tubule epithelial cells [2–4]. However, there is still no consensus on the underlying mechanism of the beneficial effect of vitamin D.

The vitamin D receptor (VDR) could be found in many organs including kidney. The VDR family is a member of a nuclear receptor family that plays a vital role in synthesis and secretion of PTH and certain proteins involved in mineral metabolism. By binding to the VDR, the kidney could regulate reabsorption of calcium and phosphate and also mediate the synthetic of vitamin D [5].

Previous study revealed that the overexpression of mitochondrial uncoupling protein 2 (UCP2) had tight positive correlation with increased proliferation, tumorigenesis, and metabolic alterations in cancer cells. In addition, UCP2 is implicated in glucose sensing systems, controlling intracellular oxidative stress, and abundance in tubular cells of the kidneys in mice and rats. In human biopsies, UCP2 is found to be mainly localized in proximal convoluted tubule cells [6]. In search of the role of vitamin D in protecting diabetic nephropathy, we determined to evaluate how VD effect on high glucose levels treated human renal tubular epithelial cells.

## 2. Methods and Materials

### 2.1. Cell Culture and Cell Transfection

Human kidney renal tubular epithelial cells (HK)-2 were purchased from ATCC (Manassas, VA, US). The cell line was cultured in DMEM/F12 medium (Gibco, Life Technologies, Bleiswijk, Netherlands) containing 10% fetal bovine serum (FBS) at 37°C/5% CO2. Chemically synthesized siRNAs (Invitrogen, Shanghai, China) were transfected into cultured HK2 cells with Lipofectamine 2000 (Invitrogen, Carlsbad, CA) for 6 h and then cultured in new medium. The siRNA sequences for human VDR were as follows: sense, 5′-GGCTGCAAAGGCTTCTTCA-3′; antisense, 5′- TTGGAAATCATTCAGCAGG-3′. The oligo sequences for human nonsense control siRNA were as follows: sense, 5′-UUCUCCGAACGUGUCACGUTT-3′; antisense, 5′-ACGUGACACGUUCGGAGAATT-3′. When cells reached 80% confluence, they were treated with normal glucose (NG group: 5.5 mmol/L), osmotic control (OC group: 5.5 mmol/ L D-glucose+24.5 mmol/ L D-mannitol), NAC control (NAC group: 30 mmol /L D-glucose + 1. 0 mmol/L N-Methylcysteine), high glucose (HG group: 30 mmol/l), HG+VD (1 × 10^−7^ mmol/l 1,25-(OH)2 D3), HG+VD+siVDR, HG+VD+AI (LY294002 10*μ*mol/l), and HG+VD+Gelipin (Gelipin 10*μ*mol/l) for 24 h. LY294002 and Gelipin were added 30 min before interfering for 24 h.

### 2.2. ELISA Assay

Activity of SOD and MDA was measured by Total Superoxide Dismutase Assay Kit with NBT and Lipid Peroxidation MDA Assay Kit (Beyotime, China). The assay was performed following manufacturer's instructions and repeated twice. The OD 450 was read on a microplate reader.

### 2.3. Flow Cytometry

Cells were digested by trypsin, and digestion was stopped by DMEM/F12 medium; after cells were collected, they were added to 15 ml mixed liquid in centrifuge tubes and centrifuged for 5 min at 8000 rpm. The supernatant was discarded, and PBS was used twice to wash the cells. The cells were incubated with ROS (MA5-26760, Life technologies), JC-1 antibody (ab141387, Abcam) and Annexin V/PI(556547, BD)at room temperature for 30 min, and flow cytometry analysis was conducted (FC500, Beckman Coulter, USA). The experiment was repeated 3 times.

### 2.4. Western Blot

Cells were harvested to isolate total protein. HK2 cells were lysed with ice-cold lysis buffer containing protease inhibitors (RIPA, Beyotime, P0013B). Proteins were resolved on 10% Tris-glycine gels and transferred to a nitrocellulose membrane. After blocking with nonfat milk for 1 h, the membrane was incubated with the primary antibody VDR Monoclonal Antibody (MA1-710, Life Technologies), Phospho-AKT1 (Ser473) Monoclonal Antibody (OMA1-03061, Life Technologies), and UCP2 Antibody (PA5-36383, Life Technologies) at a dilution of 1:1000, 4°C overnight. Membranes were washed three times and then incubated with the peroxidase-conjugated secondary antibody at a dilution of 1:2000. The ECL advanced system (GE Healthcare, Little Chalfont, UK) was used to detect the proteins.

### 2.5. Quantitative Reverse Transcription PCR

The cell lysates were harvested and extracted for total RNA using Trizol LS reagent (Invitrogen, Carlsbad, CA). The VDR, p-AKT, and UCP2 mRNA levels were evaluated with a qRT-PCR system (ABI Prism H 7700) using a 2^-△△Ct^ algorithm. The primer sequences were as follows: VDR, forward, 5′-TACAGCATCCAAAAGGTCATTG-3′; reverse, 5′-ACGCGGTACTTGTAGTCTTGGT-3′; AKT, forward, 5′-TGC ATT GCC GAG TCC AGA A-3′; reverse, 5′-GCA TCC GAG AAA CAA AAC ATC A-3′ UCP2, forward, 5′-TCTCATCACCTTTCCTCTGGA-3′; reverse, 5′-ATGGTCTTGTAGGCATTGACG-3′; GAPDH, forward, 5′-TGTTGCCATCAATGACCCCTT-3′; reverse, 5′-CTCCACGACGTACTCAGCG-3′.

### 2.6. Statistical Analysis

Statistical analyses were conducted using the software GraphPad Prism 5.0 and SPSS 15.0. Data are expressed as the mean±SD. Analysis of variance (ANOVA) was used to evaluate significant differences among the groups and Tukey's test was used as post hoc test. A p value <.05 was considered as statistically significant.

## 3. Results

### 3.1. Activity of SOD and MDA

**Figure 1:**
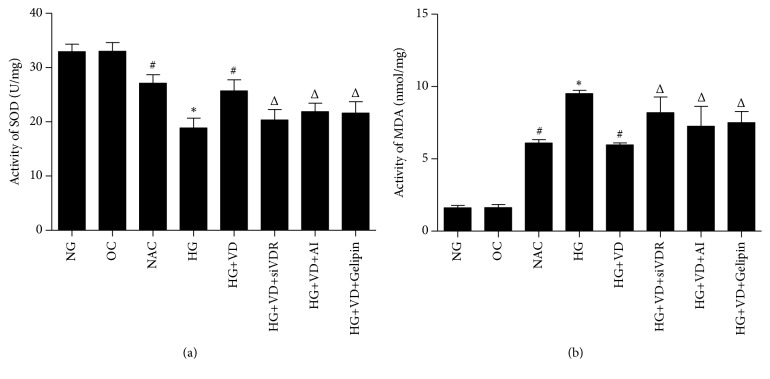
*Change of activity of SOD (a) and MDA (b) in human tubular epithelium cell line (HK2) using ELISA assay.* Data are presented as mean±SD. *∗*P <0.01 versus NG group; #P <0.01 versus HG group; ΔP <0.01 versus HG+VD group.

In order to investigate the change of oxidative stress, ELISA assay for activity of SOD and MDA was performed. As shown in Figure 1, high glucose significantly alleviated the activity of SOD and elevated the activity of MDA, compared to NG and OC group (*p*<0.01). In HG+VD group, the activity of SOD was significantly higher than that in HG group (*p*<0.01), while the activity of MDA was significantly lower than that in HG group (*p*<0.01), suggesting that vitamin D obviously reversed the effect of high glucose. siRNA, AI, and Gelipin treatment significantly decreased the activity of SOD and increased the activity of MDA compared to HG+VD and NAC group (*p*<0.01).

### 3.2. ROS, Mitochondrial Membrane Potential, and Apoptosis of the HK2 Cells

**Figure 2:**
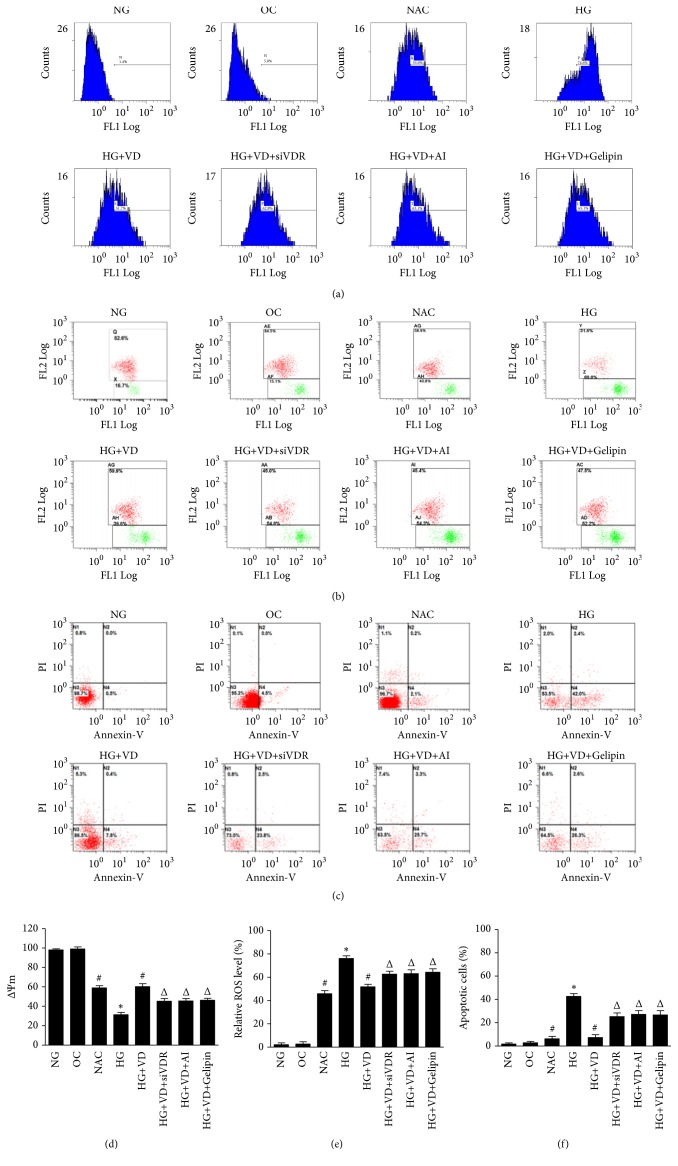
*Flow cytometry analysis for ROS, mitochondrial membrane potential, and apoptosis.* (a) Representative flow cytometric figures of cells were presented in (a), (b), and (c). (d), (e), and (f) calculated the relative level of mitochondrial membrane potential, ROS, and apoptosis. The data are expressed as the mean±SD from three independent experiments. *∗*P <0.01 versus NG group; #P <0.01 versus HG group; ΔP <0.01 versus HG+VD group.

To assess the effect of VD, we examined the HK2 cells for ROS, mitochondrial membrane potential, and apoptotic events. Decrease of mitochondrial membrane potential represents the early apoptosis of cells. As shown in Figure 2, the data of ROS and JC-1 revealed that high glucose caused significant oxidative stress and decreased mitochondrial membrane potential of HK2 compared to NG group (*p*<0.01). Addition of siVDR, AI, and Gelipin significantly increased the oxidative stress and inhibited mitochondrial membrane potential when compared to HG+VD and NAC group (*p*<0.01).

Apoptosis in HD+VD group was significantly lower than that in HG group (*p*<0.01). Apoptosis in HG+VD+siVDR, HG+VD+AI, and HG+VD+Gelipin was significantly higher than that in HG+VD group (*p*<0.01).

### 3.3. The Role of AKT/UCP2 Signaling Pathway

**Figure 3:**
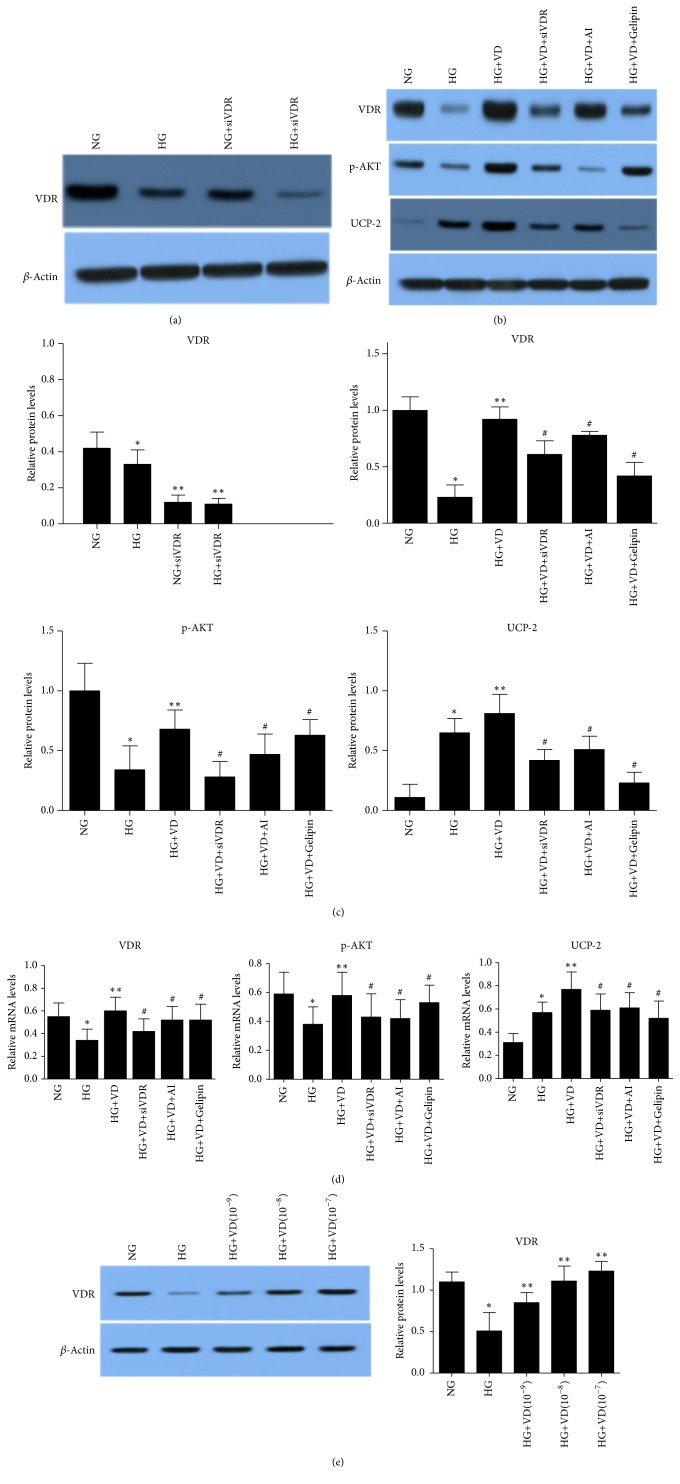
*Protein and mRNA levels of HK2 cells analyzed by western blot and RT-PCR.* (a), (b), and (c) show the protein levels of VDR, p-AKT, and UCP2 evaluated by western blot. (d) mRNA levels of VDR, AKT, and UCP2 measured by RT-PCR. Results are presented as the fold change in activity relative to normoxic cells. The data are expressed as the mean ± SD. *∗p*<0.01 vs NG, *∗∗p*<0.01 vs HG, #*p*<0.01 vs HG+VD.

To determine the existence and dominant role of VDR, we performed western blot and RT-PCR. Results showed VDR protein and mRNA levels in NG+siVDR group were significantly lower than those in NG group (Figure 3(a),* p*<0.01). And VDR protein and mRNA levels in HG+ siVDR group were significantly lower than those in HG group (*p*<0.01). Western blot assay showed HG+VD (10^−9^, 10^−8^, 10^−7^) significantly increased the VDR levels compared to those in HG group (Figure 3(e)).

To verify the molecular mechanism of vitamin D in protecting HK2 cells from high glucose induced apoptosis, we monitored the activation of stress response pathways in HK2 cells. As can been seen in Figure 3(b), VDR and p-AKT protein levels in HG group were significantly lower than those in NG and HG+VD group (*p*<0.01). UCP2 protein levels in HG group were significantly higher than those in NG group (*p*<0.01). VDR, p-AKT, and UCP2 protein levels in HG+VD group were significantly higher than those in HG group (*p*<0.01). However, VDR, p-AKT, and UCP2 protein levels under interference with siVDR, AI, and Gelipin significantly decreased compared to HG+VD group (*p*<0.01).

As shown in Figure 3(d), VDR and AKT mRNA levels in HG group were significantly lower than those in NG group (*p*<0.01) while UCP2 mRNA levels in HG group were significantly higher than those in NG group (*p*<0.01). VDR, AKT, and UCP2 mRNA levels in HG+VD group significantly increased when compared with HG group (*p*<0.01), whereas VDR, AKT, and UCP2 mRNA levels in HG+VD+siVDR, HG+VD+AI, and HG+VD+Gelipin group significantly decreased compared to HG+VD group (*p*<0.01).

## 4. Discussion

It is known that SOD and MDA play a crucial role in the balance of oxidation and antioxidant function of the body, and SOD activity can reflect the free radical scavenging ability of the body directly. To our knowledge, elevation of ROS (e.g., •O^2−^, •OH) can cause oxidative stress. Glucose exposure attenuates the antioxidant and generates ROS in many cell types. Accumulating evidence showed high glucose treatment was able to induce ROS production and break the balance of oxidation and antioxidant, resulting in apoptosis of podocyte [7, 8]. Here, we found a decrease of SOD and increase of MDA and apoptosis induced by high glucose in human tubular epithelium cell, which was reversed by vitamin D. The results showed that vitamin D has the ability to inhibit HG-induced oxidative stress, mitochondrial fission, and ROS generation. Nevertheless, the protective effect was prevented by three inhibitors: siVDR, AI, and Gelipin.

Considering all biological actions of vitamin D and synthetic analogs mediated by the binding to the VDR, we investigated the activity of VDR in HK2 cells. We observed the significant fall of VDR protein and mRNA in high glucose concentration medium cultured HK2 cells, which was in accordance with previous researches.

Interestingly, synthetic vitamin D analogs were confirmed to inhibit the growth of mammary tumors via regulating the AKT signaling pathways [9]. AKT, an ROS target, plays a dominant role in maintaining cell growth and proliferation, and the downregulation of its activity contributes to renal proximal tubular cell apoptosis [10, 11]. Moreover, the mitochondrial carrier UCP2 can regulate oxidative stress, mitochondrial membrane potential (△Ψm), and energetic metabolism [12]. In* in vivo* and* in vitro* models of ischemic acute kidney injury, there was evidence that UCP2 was upregulated and helped renal tubular cells to survive [13]. To verify the mechanism of the efficacy of the ligands for VDR, we demonstrated the activation of AKT and UCP2 in downstream of ROS. In this study, we found that a rise of UCP2 and alleviation of VDR and p-AKT in HG-induced HK2 cells confirmed the damage caused by HG. We found that VD enhanced VDR, p-AKT, and UCP2 expression in protein and gene levels in HG-induced cells, partially attenuated by siVDR, AI, and Gelipin, suggesting the involvement of activation of AKT and UCP2.

In conclusion, we have shown that VD was able to prevent high glucose concentration induced oxidative stress and apoptosis in human tubular epithelium cells. These observations of VDR, AKT, and UCP2 inhibitor suggest that VD may play a key role in protecting human tubular epithelium cells in a VDR dependent AKT/UCP2 signaling pathway.

## Data Availability

The data used to support the findings of this study are included within the article.
